# An evaluation of the use of inhalers in asthma and chronic obstructive pulmonary disease

**DOI:** 10.1016/j.jtumed.2023.01.001

**Published:** 2023-01-12

**Authors:** Seçil Çakmaklı, Ayşe Özdemir, Hikmet Fırat, Cenk Aypak

**Affiliations:** a Department of Family Medicine, University of Health Sciences, Ankara Dışkapı Yıldırım Beyazıt Training and Research Hospital, Ankara, Turkey; b Department of Pulmonary Diseases, University of Health Sciences, Ankara Dışkapı Yıldırım Beyazıt Training and Research Hospital, Ankara, Turkey

**Keywords:** الربو, مرض الانسداد الرئوي المزمن, استخدام الأدوية, أجهزة الاستنشاق المقننة الجرعة, بخاخات المسحوق الجاف, Asthma, Chronic obstructive, Drug misuse, Dry powder inhalers, Metered dose inhaler, Pulmonary disease

## Abstract

**Objectives:**

Inhaled therapy is the treatment of choice for obstructive lung diseases such as asthma and chronic obstructive pulmonary disease (COPD). However, the maximum benefit from such therapy depends on the correct use of inhaler devices. In this study, our primary aim was to evaluate inhaler techniques in patients with asthma and COPD in order to identify common errors. In addition, we investigated the effect of various parameters on the rate of inhaler misuse.

**Methods:**

We enrolled a total of 300 asthma/COPD patients, who presented at the Chest Diseases and Family Medicine Outpatient Clinics of a tertiary hospital located in Ankara, Turkey. We used a face-to-face survey that included questions about sociodemographic features and inhaler therapy. Subsequently, we requested patients to demonstrate how they use their inhalers and assessed their inhalation technique according to checklists.

**Results:**

Of the 300 patients, 70.2% used their inhaler drugs incorrectly. The rate of misuse among metered dose inhaler (MDI) users was significantly higher than those using dry powder inhalers (DPIs) (77.6% vs 64%; p = 0.002). When DPI devices were analyzed, the rates of misuse were significantly higher in Handihaler users (p = 0.012) and Diskus inhaler users (p = 0.009) when compared to Sanohaler users. Gender, type of disease (asthma/COPD), duration of inhaler use, and duration of illness had no impact on the rate of misuse. However, an advanced age (>60 years old), a level of education lower than high school, and the use of MDI were all identified as factors associated with misuse. The most common mistake was ‘failing to breath out before inhalation’ for all types of devices (for MDI: 66.7%, and for DPI: 71.1–82.8%).

**Conclusions:**

The rate of inhaler drug misuse was high. The identification of factors associated with misuse could provide information to implement appropriate actions to reduce the rates of misuse.

## المخلص

**أهداف البحث:**

العلاج بالاستنشاق هو العلاج المفضل لأمراض الرئة الانسدادي مثل الربو ومرض الانسداد الرئوي المزمن. ومع ذلك، فإن أقصى فائدة من العلاج تعتمد على الاستخدام الصحيح لأجهزة الاستنشاق. في هذه الدراسة، كان هدفنا الأساسي هو تقييم تقنيات استخدام أجهزة الاستنشاق لدى مرضى الربو ومرض الانسداد الرئوي المزمن من أجل تحديد الأخطاء. أيضا، قمنا بفحص تأثير المعلمات المحتملة على معدل إساءة الاستخدام.

**طرق البحث:**

تم تسجيل ما مجموعه 300 من مرضى الربو / مرض الانسداد الرئوي المزمن، الذين قدموا عيادات أمراض الصدر وطب الأسرة للمرضى الخارجيين في مستشفى ثالث يقع في أنقرة - تركيا ، في الدراسة. استخدمنا استطلاعًا وجهاً لوجه تضمن أسئلة حول الميزات الاجتماعية والديموغرافية والعلاج بالاستنشاق. بعد ذلك، طلبنا من المرضى توضيح كيفية استخدامهم لأجهزة الاستنشاق وتقييم تقنية الاستنشاق الخاصة بهم بسبب قوائم المراجعة.

**النتائج:**

من بين جميع المرضى، كان 70.2٪ يستخدمون أدوية الاستنشاق بشكل غير صحيح. كان معدل إساءة الاستخدام بين مستخدمي أجهزة الاستنشاق بالجرعات المقننة أعلى مقارنة بمستخدمي أجهزة الاستنشاق بالمسحوق الجاف (77.6٪ مقابل 64٪). عند مقارنة أجهزة الاستنشاق بالمسحوق الجاف فيما بينها، كانت معدلات إساءة الاستخدام أعلى بشكل ملحوظ في مستخدمي هانديهالر ومستخدمي أجهزة الاستنشاق ديسكس مقارنة بمستخدمي سانوهالر. تم العثور على الجنس ونوع المرض (الربو / مرض الانسداد الرئوي المزمن) ومدة استخدام جهاز الاستنشاق ومدة المرض ليس لها أي تأثير على سوء الاستخدام. ومع ذلك، فإن العمر المتقدم (> 60 سنة)، ومستوى التعليم الأدنى من المدرسة الثانوية، واستخدام أجهزة الاستنشاق بالجرعات المقننة كانت من العوامل المرتبطة بسوء الاستخدام. كان الخطأ الأكثر شيوعا هو "عدم التنفس قبل الاستنشاق" لجميع أنواع الأجهزة (بالنسبة إلى أجهزة الاستنشاق بالجرعات المقننة: 66.7٪ ، و أجهزة الاستنشاق بالمسحوق الجاف: 71.1-82.8٪).

**الاستنتاجات:**

تم العثور على معدل إساءة استخدام العقاقير المستنشقة لتكون عالية. من شأن تحديد العوامل المرتبطة بسوء الاستخدام أن يوفر عناصر لتنفيذ الإجراءات لتقليل معدلات فشل العلاج.

## Introduction

Asthma and chronic obstructive pulmonary disease (COPD) are increasingly prevalent respiratory diseases that are associated with chronic inflammation of the airways and can cause airway obstruction.^1^^,^^2^ It is estimated that asthma affects 300 million individuals worldwide with a global prevalence of 1–16%; the global prevalence of COPD has been estimated to 11.7%.^3^^,^^4^ Both diseases require long-term treatment on a regular daily basis.^2^^,^^3^

Inhaled therapy is the cornerstone of pharmacological treatment for COPD and asthma and has many advantages such as a limited number of adverse effects, small doses of medication, rapid onset of action, and reduced systemic absorption.^4^, ^5^, ^6^ However, despite these advantages, treatment with inhaler devices is more expensive than oral and parenteral therapy and the patients involved should be taught inhalation techniques that are necessary for the use of these devices. Furthermore, there are more than 250 commercially available inhaler devices (traditionally distinguished as pressurized metered dose inhalers (MDIs) and dry powder inhalers (DPIs)). Furthermore, each device has its own operating mode. Although these inhalers are used in a similar manner, it is not easy to learn how to use these devices.^6^

The most important and crucial factor with regards to the efficiency of treatment is that the patient uses the inhaler in the correct manner so as to optimize the therapeutic response.^2^ The use of inhaler devices can be challenging; this can result in critical errors in handling that significantly reduce the delivery of drugs into the lungs, thus increasing the risk of potential adverse effects.^7^ The inability to use inhalers, the misuse of devices, and poor compliance with inhaler therapy, are frequently observed among patients.^5^ The misuse of inhaler devices can lead to undesirable consequences such as the prevention of disease control; it can also have negative effects on patient confidence and contributes to the wasting of resources.

In this study, our primary aim was to evaluate the rate of inhaler misuse and to investigate the effects of key parameters such as age, gender, educational level, device type, and the duration of use, on the misuse of inhaler devices in patients with asthma and COPD who had been prescribed inhalation therapy. Our secondary aim was to identify the specific errors that led to misuse in order to reduce the rates of treatment failure.

## Materials and Methods

### Study design and setting

The study was designed as a cross-sectional and descriptive study incorporating a cohort of patients treated and followed up due to asthma and/or COPD at the Family Medicine and Pulmonary Diseases Outpatient Clinics of Diskapi Yildirim Beyazit Research and Training Hospital (Ankara/Turkey).

### Sample size

Approximately 1300 patients with asthma and COPD were referred to the Family Medicine and Pulmonary Diseases Outpatient Clinics of our hospital within the three months of study. Power calculation determined that the sample size should be at least 297 with a 95% confidence level and a 5% margin of error.

### Patients

We enrolled a total number of 300 patients aged over 18 years, who had been using inhaler devices for at least 6 months. Patients who were not cooperative or orientated, who had language or psychiatric problems that would affect their ability to answer questions, those who were receiving inhalation therapy for the first time, and those who used inhalers for the treatment of diseases other than asthma or COPD, were excluded from the study.

### Data collection tool

A pre-prepared questionnaire was administered to the patients by a face-to-face interview technique. Participants provided informed consent before participating in the study. We recorded a range of patient demographic data, including age, gender, educational level, and diagnosis. In addition, we surveyed each patient with regards to the following issues: the duration of inhaler device use, how they learned to use their inhaler devices, the first healthcare provider (HCP) prescribing the medication, the reasons why the family physicians prescribed their medication or provided consultation for inhaler treatments, satisfaction with their treatment, difficulties associated with the use of inhaler devices, views on side effects such as addiction and weight gain, opinions about educational videos on the internet/social media and their usefulness. Afterward, the patients were asked to administer medication using their own inhaler device, and the inhalation procedure was observed. We modified the National Asthma Council Australia's checklists for healthcare professionals to prepare our checklists in order to determine specific errors.^8^ The patient's ability to use their inhaler device was evaluated according to these specific checklists (presented in Table 1); all errors were recorded. The inhaler skills of patients who made major mistakes were classified as ‘incorrect use’ and errors in non-major steps of the checklists were classified as ‘inadequate use’. Both incorrect use and inadequate use were classified as instances of misuse.

**Table 1:** Checklists for inhaler devices.

MDI^a^	Single dose DPI^b^	Discus	Turbuhaler	Handihaler	Sanohaler
1. Check the device whether there is adequate drug in it (by shaking or visually)	**1. Remove the cap**	1. Check the device visually whether there is adequate drug in it	**1. Unscrew and remove cover**	1. Remove the cap	1. Check the device visually whether there is adequate drug in it
**2. Remove the cap**	**2. Open mouthpiece correctly**	**2. Turn the protective cover by placing your thumb in the slot and turning it**	2. Check the device visually whether there is adequate drug in it	2. Flip open mouthpiece	**2. Remove the cap**
3. Hold the inhaler device upright and shake well	**3. Place capsule in chamber and close**	3. Click the device to open it and make sure it is inserted into the slot (Hold horizontally, load dose by sliding lever until it clicks)	**3. Twist the grip of the inhaler**	3. Place capsule in chamber and close	**3. Press the button**
4. Breath out fully and gently	**4. Explode the capsule**	**4. Prepare the medicine for use by pushing the latch.**	**4. Hear the click (pop it)**	**4. Explode the capsule**	4. Breath out fully and gently
5. Keep device in proper position	5. Breath out fully and gently	5. Breath out fully and gently	5. Breath out fully and gently	5. Breath out gently	5. Keep device in proper position
6. Put mouthpiece between teeth without biting and close lips to form a good seal	6. Keep device in proper position	6. Keep device in proper position	6. Keep device in proper position	6. Keep device in proper position	6. Put mouthpiece between teeth without biting and close lips to form a good seal
**7. Keep breathing in slowly and deeply and press 1 time**	7. Put mouthpiece between teeth without biting and close lips to form good seal	7. Put mouthpiece between teeth without biting and close lips to form a good seal	7. Put mouthpiece between teeth without biting and close lips to form a good seal	7. Put mouthpiece between teeth without biting and close lips to form a good seal	**7. Breathe in quickly and steadily**
8. While holding breath, remove inhaler from mouth	**8. Breathe in quickly and steadily**	**8. Breathe in quickly and steadily**	**8. Breathe in quickly and steadily**	**8. Breathe in quickly and steadily**	8. While holding breath, remove inhaler from mouth
9. Hold your breath for 8–10 s	9. While holding breath, remove inhaler from mouth	9. While holding breath, remove inhaler from mouth	9. While holding breath, remove inhaler from mouth	9. While holding breath, remove inhaler from mouth	9. Hold your breath for 8–10 s
	10. Hold your breath for 8–10 s	10. Hold your breath for 8–10 s	10. Hold your breath for 8–10 s	10. Hold your breath for 8–10 s	
	11. Open mouthpiece and check that the capsule is completely empty			11. Open mouthpiece and check that the capsule is completely empty	
	12. If more than one dose is needed, repeat the steps				

The list of the steps for the correct use of different inhaler devices. The steps that were evaluated as major errors are written in **bold**. While the inhaler usage skills of the patients who made major mistakes were evaluated as ‘incorrect use’, the errors at non-major steps of the checklists were recorded as ‘inadequate use’. Both incorrect use and inadequate use mean misuse of inhalers.

a Metered Dose Inhaler.

b Dry Powder Inhaler.

Following the assessment of inhaler skills, the same investigator described the correct use of the inhaler device to the patients during face-to-face training sessions. These training sessions lasted at least 5 min. In addition, appropriate device setup and inhalation techniques were described by using demonstration devices. We did not use brochures or video demonstrations. All surveys, educational interventions/training were carried out by a single investigator.

### Statistical analysis

The data obtained in this study were statistically analyzed using the IBM SPSS Statistics for Windows v.20.0 statistical package software (IBM Corp., Armonk, NY). Normality, statistical interactions, and collinearity were assessed using the Kolmogorov–Smirnov test. Using descriptive analysis techniques, all continuous variables were computed as mean and standard deviation (SD) or as median and interquartile range (IQR). Categorical variables were described as frequency and percentage. In accordance to meeting the assumptions, Chi-squared or Fisher's Exact Tests were applied to determine the association between categorical variables. The dependent variable in this study was inhaler usage classified as either misuse or correct use. Independent risk factors (age, education level, and type of inhaler) were analyzed by logistic regression analysis, and odds ratios (OR) were calculated with 95% confidence intervals (CI). A p-value of ≤0.05 was considered statistically significant.

## Results

We recruited 300 patients (mean age: 58.1 ± 13.2 years): 187 with asthma (62.3%) and 113 with COPD (37.7%). The median duration of inhaler therapy was 4 years (IQR: 2).

In total, 127 patients were using more than one inhaler device. For these patients, we assessed the inhaler technique for each device separately; thus, a total of 440 inhaler devices were assessed. Almost all patients (98.7%; n = 296) were able to recognize the inhaler devices they used when shown a choice of demonstration devices and 28.3% (n = 85) could correctly name their inhaler drugs. The most common device type was the MDI (67%). Analysis showed that 88% of the enrolled patients received their first training on inhaler use from a pulmonologist. A total of 1.7% of cases reported that they did not receive any training with regards to the use of inhalers (Table 2).

**Table 2:** Characteristics of the study population.

Characteristic	n (%)
**Gender**	
Male	114 (38)
Female	186 (62)
**Type of disease**	
Asthma	187 (62.3)
COPD	113 (37.7)
**Educational status**	
Illiterate	60 (20)
Literate	18 (6)
Primary school	157 (52.3)
Secondary school	19 (6.3)
High school	32 (10.7)
College	14 (4.7)
**Inhaler devices**^a^	
MDI^b^	201 (67)
Single dose DPI^c^	97 (32.3)
Discus/discair	55 (18.3)
Turbuhaler	26 (8.7)
Handihaler	41 (13.7)
Sanohaler	20 (6.7)
**Source of information about inhaler usage**	
Pulmonologist	264 (88)
Pharmacist	14 (4.2)
Internist	6 (2)
Family physician	5 (1.7)
Other specialists	3 (0.9)
Brochure/leaflet	1 (0.3)
Nobody described	5 (1.7)
Prospectus	2 (0.7)

a One hundred twenty-seven patents were using more than one inhaler device, the technique employed to use the inhaler assessed for each device respectively; thus, a total of 440 inhaler devices were assessed.

b Metered Dose Inhaler.

c Dry Powder Inhaler.

We also assessed the opinions of patients with regards to the side effects of inhaler drugs and the necessity for educational videos on inhaler devices. Subsequently, we recorded their status with regards to watching educational videos about inhaler devices on the internet/social media (Table 3).

**Table 3:** Patient opinions on the side effects of inhaler drugs and training media.

Patient opinions on inhaler drugs	n	%
***Patient opinions about the side effects of inhaler drugs***		
Does inhaler medication/treatment cause addiction?		
Yes	36	12
No	102	34
Don't know	162	54
Does inhaler medication cause weight gain?		
Yes	57	19
No	122	40.7
Don't know	121	40.3
***Watching inhaler training videos***		
Have you ever watched educational/training videos about using inhaler devices on the internet/social media?		
Yes	22	7.3
No	278	92.7
***Patient opinions about the necessity for video training programs on the internet/social media***		
Are video training programs on the internet and social media necessary?		
Absolutely necessary	33	11
Necessary	135	45
Not necessary/no need to	41	13.7
No idea/Don't know	91	30.3

Only 29.8% (n = 131) of all inhaler devices (n = 440) were used correctly by the patients. When considering all devices, the rate of misuse was 70.2%. There was no significant difference in age when compared between misusers and correct users (vs 56.1 ± 11.8; p = 0.089). In this study, we investigated the effect of inhaler device type on misuse. The rate of misuse was significantly higher among MDI users when compared to DPI users (77.6% vs 64%; p = 0.002). When the use of MDI was compared with types of DPI devices; the rate of misuse was significantly higher for MDI users when compared to single capsule inhalers (p = 0.002) and the Sanohaler (p < 0.001). When DPI devices were compared between themselves, the rate of misuse was lowest among the Sanohaler users (40%). The correct use of inhalers was significantly higher in the ≤60 years group than the >60 years group (28.3% vs 17.9%; p = 0.035). The rate of correct use was also significantly higher in patients with a higher level of education. The rate of misuse was 79.5% among patients with an educational level below high school but was 58.7% in patients with an educational level of high school or higher (p = 0.002). No significant difference in the correct use of inhalers was observed between genders, type of disease/diagnosis (asthma/COPD), duration of inhaler use/therapy, and time since the diagnosis (Table 4).

**Table 4:** Comparison of clinical variables between groups.

Variables	Correct use n (%)	Misuse n (%)	p
**Age (year)**			
≤60	50 (30.1)	116 (69.9)	**0.046**
>65	28 (20.9)	106 (79.1)	
**Gender**			
Men	31 (27.2)	83 (72.8)	0.786
Women	47 (25.3)	139 (74.7)	
**Type of disease**			
Asthma	40 (21.4)	147 (78.6)	0.233
COPD	31 (27.4)	82 (72.6)	
**Inhaler device usage time (year)**			
<1	9 (25)	27 (75)	0.893
1–5	36 (24.5)	111 (75.5)	
>5 year	26 (22.2)	91 (77.8)	
**Number of inhaler devices each patient use**			
1	46 (26.6)	127 (73.4)	0.164
>1	25 (19.7)	102 (80.3)	
**Type of inhalers**			
MDI^a^	45 (22.4)	156 (77.6)	**0.002**
Single dose DPI^b^	38 (39.2)	59 (60.8)	
Discus/discair	15 (27.3)	40 (72.7)	
Turbuhaler	10 (38.5)	16 (61.5)	
Handihaler	11 (26.8)	30 (73.2)	
Sanohaler	12 (60)	8 (40)	
**Recognition the inhaler**			
Yes	71 (24)	225 (76)	0.576
No	0 (0)	4 (100)	
**Knowing the name of the inhaler**			
Yes	22 (25.9)	63 (74.1)	0.570
No	49 (22.8)	166 (77.2)	
**Drug satisfaction**			
Yes	53 (23.8)	170 (76.2)	0.442
No	2 (11.8)	15 (88.2)	
Partially	16 (26.7)	44 (73.3)	
**Internet/social media usage for inhaler education**			
Yes	9 (40.9)	13 (59.1)	**0.048**
No	62 (22.3)	216 (77.7)	
**Necessity of educational videos**			
Absolutely necessary	13 (39.4)	20 (60.6)	**0.043**
Necessary	34 (25.2)	101 (74.8)	
Unnecessary	10 (24.4)	31 (75.6)	
No idea	14 (15.4)	77 (84.6)	

a Metered Dose Inhaler.

b Dry Powder Inhaler.

The patients who participated in our study were compared according to the correct or incorrect (misuse) of inhaler devices in terms of recognizing the drug when seeing it, knowing the name of the drug, being satisfied with the drug, following education on the internet and social media, and seeing training as being necessary for the use of inhaler devices. We found that the rate of misuse for inhaler devices was low in those who watched educational material on the internet and social media (p = 0.048). The rate of misuse for inhaler devices was significantly lower in those who thought that education on the internet and social media was absolutely necessary (p = 0.043) (Table 4).

The distribution of errors was very similar and the most common error was failure to breathe out before inhalation; this was the case for all types of devices. Other frequent mistakes included the patient not shaking the MDI or not holding their breath after inhalation. The most common mistakes and the error rates associated with different inhalers are shown in Table 5.

**Table 5:** The most common mistakes according to different types of inhaler.

Device	Mistakes	n	%
MDI^a^	Not breathing out before inhalation	100	66.7
	Not shaking the device	75	50
	Not breath holding after inhalation (8–10 s)	73	48.7
Single Dose DPI^b^	Not breathing out before inhalation	45	78.9
	Not breath holding after inhalation (8–10 s)	36	63.2
	Not controlling whether some powder drug rests into the capsule after inhalation	3	5.3
Discus/Discair	Not breathing out before inhalation	27	7.1
	Not breath holding after inhalation (8–10 s)	21	55.3
	Not checking the device visually whether there is adequate drug in it	16	31.2
Turbuhaler	Not breathing out before inhalation	12	80
	Not breath holding after inhalation (8–10 s)	11	73.3
	Not checking the device visually whether there is adequate drug in it	3	20
Handihaler	Not breathing out before inhalation	24	82.8
	Not breath holding after inhalation (8–10 s)	17	58.6
Sanohaler	Not checking the device visually whether there is adequate drug in it	5	18.5
	Not breathing out before inhalation	6	75
	Not breath holding after inhalation (8–10 s)	3	37.5

a Metered Dose Inhaler.

b Dry Powder Inhaler.

Logistic regression analysis was performed to determine independent parameters that might affect the rate of misuse for inhaler devices. Univariate analysis indicated that age, education and the type of inhaler device were significant factors with regards to the rate of inhaler misuse; being over 60 years-of-age increased the risk of misuse by 1.64-fold (p = 0.044). A level of education lower than high school increased the risk of misuse by 2.49-fold (p = 0.010) while the use of MDI increased the risk of misuse by 3.74-fold (p < 0.001) (Table 6).

**Table 6:** Logistic regression analysis.

Parameter	OR^a^ (95%I^b^)	p
>60 years of age (ref: ≤60 years of age)	**1.64** (0.93–2.81)	**0.044**
High school and below (ref: high school and above)	**2.49** (1.23–5.00)	**0.010**
Metered Dose Inhaler (ref: Dry Powder Inhaler)	**3.74** (2.16–6.46)	**<0.001**

a Odds ratio.

b Confidence interval.

## Discussion

The incorrect use of inhalers can lead to inadequate adherence, poor asthma and COPD control, patient dissatisfaction, and even economic burden. The misuse of inhalers has been an obstacle to both patients and clinicians for many years and unfortunately still remains a significant issue. Therefore, we examined the rates of inhaler misuse and the factors that might be involved.

Previous studies found that patient age, gender, educational status, the duration of disease, the type of inhaler device, correct inhalation technique, and the use of multiple devices, all played a role in the effectiveness of the drugs used by inhalation.^9^, ^10^, ^11^, ^12^, ^13^ Although old age was considered as a risk factor for inhaler misuse, specific data pertaining to this issue were debatable.^13^ Young patients can be expected to use inhaler devices or any other medications more accurately due to higher levels of cognitive function. However, a significant relationship between age and the misuse of inhalers has yet to be clearly demonstrated. For instance, Pessôa et al. found that inhaler misuse was higher in patients older than 50 years-of-age, but without a statistically significant difference.^10^ Furthermore, a recent systematic review showed that patients who were older and younger than 65 years significantly misused their inhalers; in addition, they made the same types of mistakes.^11^ In contrast, we found that the rate of misuse was higher in patients older than 60 years-of-age. In many studies, gender was not found as a factor that would make a significant difference, such as age.^10^, ^11^, ^12^ Consistent with the literature, we found that the rate of misuse was higher in patients older than 60 years-of-age and found no difference between genders in terms of the technique used with inhalers.

Previous studies found no significant relationship between educational level and the rate of inhaler misuse, although their study populations focused on different levels of education. A study conducted in Taiwan showed that the majority of patients who participated had low educational levels; no relationship was detected between the patient's education level and the rate of inhaler misuse.^13^ Similarly, Pothirat et al. investigated 108 patients (73.1% were highly educational) and found that the rates of misuse differed when compared between the low-education and the high-education groups.^12^ In our study, the rates of misuse were higher in patients with an educational level of high school and below.

Video and multimedia or internet-based education programs have contributed to the increasing success rate in inhaler technical education.^12^ Park et al. compared video education and face-to-face education for asthma patients and showed that video education may be a useful substitute for face-to-face education.^14^ Therefore, it would be a rational approach to use the power of media to increase awareness about COPD, asthma and appropriate treatments. In our study, we asked our patients whether they had watched educational videos previously; regardless of their answer to this question, we also asked their opinions on the necessity for such videos. The rates of misuse for inhaler devices were lower in patients who had watched educational videos and/or those who thought that these videos were “absolutely necessary”. On the other hand, although more than half of the patients thought that training videos were necessary, the proportion of patients who had watched training videos was quite low (7.3%).

When comparing between the rates of inhaler usage in patients who were well-educated about inhaler usage and the rate of correct inhaler use, a positive correlation was detected.^13^^,^^14^

We also found that inhaler misuse led to insufficient inhaler use, poor disease control, and reduced levels of patient satisfaction.^14^ In our study, most of the patients stated that they were satisfied with their inhaler drugs, despite a high rate of misuse. This highlights that a patient's satisfaction with their inhaler does not mean that they use it correctly and effectively.

Some previous studies found that different inhaler devices may be associated with different rates of misuse. In their systematic literature review and meta-analysis, Chrystyn et al. compared different inhaler devices and stated that the rate of at least one critical error could reach 92%. Furthermore, the rates of misuse were 45.6% and 28.4% for MDI and DPI devices, respectively.^15^ In contrast, Basheti et al. found a significantly higher rate of correct inhaler technique in patients who used MDI.^16^ In the present study, we found a significant difference in the rate of misuse when comparing two inhaler groups against MDI.

This study has also several limitations that need to be considered. First, since the study was carried out in one center, our results cannot be generalized. Second, we did not analyze spirometry values and disease severity; these are important for an objective evaluation. Third, although, the patients were educated on the use of inhaler devices, the rates of misuse were not checked following education. However, this study draws attention to the steps where the most frequent mistakes were made in inhaler use. Auditing of inhaler techniques and re-education sessions were carried out by the same investigator, thus eliminating potential variations among multiple observers.

## Conclusion

The rates of inhaler misuse were high in patients with asthma and COPD. These rates were even higher in patients older than 60 years, who had an educational level of high school or below, and who were using MDI. Almost two-thirds of patients using MDI had a significantly higher rate of misuse. All clinician who deal with such patients should be aware of these factors and each patient's visit should be taken as an opportunity to evaluate the patient's inhaler use in order to correct their technique and optimize treatment.

## Source of funding

This research did not receive any specific grant from funding agencies in the public, commercial, or not-for-profit sectors.

## Conflict of interest

The authors have no conflict of interest to declare.

## Ethical approval

This study was approved by the local ethics committee of our institution (date: 10 September 2018; no: 54/15).

## Authors contributions

SÇ, CA and HF: designed the study and conducted the research; SÇ, CA and HF: collected, organized, and analyzed the data; SÇ, AÖ and CA: wrote the initial and final drafts of the article; SÇ, AÖ, HF and CA: gave final approval of the version to be submitted. All authors have critically reviewed and approved the final draft and are responsible for the content and similarity index of the manuscript.
